# Protocol for the development of a consensus practice guideline To address clinical and regulatory barriers to buprenorphine dispensing in community pharmacy

**DOI:** 10.1186/s13690-024-01287-4

**Published:** 2024-04-25

**Authors:** Tyler J. Varisco, Hannah Fish, Joshua Bolin, David Dadiomov, Lucas G. Hill, Ekere J. Essien, Matthew A. Wanat, Diane Ginsburg, Jeanne Waggener, Sahar Yazdanfard, Juhyeon Song, Whanhui Chi, Douglas Thornton

**Affiliations:** 1 The Prescription Drug Misuse Education and Research Center, University of Houston College of Pharmacy, Houston, USA; 2 Department of Pharmaceutical Health Outcomes and Policy, University of Houston College of Pharmacy, 4349 Martin Luther King Blvd, 77204 Houston, TX USA; 3 The National Community Pharmacists Association, Alexandria, USA; 4 The National Association of Boards of Pharmacy, Mount Prospect, USA; 5 Titus Family Department of Clinical Pharmacy, The University of Southern California College of Pharmacy, Los Angeles, USA; 6 Division of Pharmacy Practice, University of Texas at Austin College of Pharmacy, Austin, USA; 7 Department of Pharmacy Practice and Translational Research, University of Houston College of Pharmacy, Houston, USA

**Keywords:** Buprenorphine, Opioid substitution treatment, Pharmacy, Delphi technique, Guidelines as topic

## Abstract

**Background:**

Less than half of community pharmacies in the United States stock buprenorphine products indicated for the treatment of opioid use disorder. This lack of access to buprenorphine in community pharmacies is a significant barrier to care. To address this issue, this protocol outlines a comprehensive approach to develop a practice guideline aimed at improving access to safe and effective opioid use disorder treatment in community pharmacies.

**Methods:**

The guideline development process will proceed in three phases, following a technique closely aligned with the Institute of Medicine’s guidance on guideline development. The first phase will involve conducting qualitative interviews with pharmacists in three states to identify their beliefs toward buprenorphine dispensing. As limitations on buprenorphine supply are related to constraints at all levels of the drug supply and regulatory system, the second phase, we will recruit representatives from regulatory agencies, pharmacy organizations, the Drug Enforcement Administration, pharmaceutical wholesalers as well as addiction medicine physicians and psychiatric pharmacists to develop consensus recommendations through a modified Delphi design. This will be followed by a public comment period and external expert review of the recommendations led by the National Association of Boards of Pharmacy. Finally, in the third phase, a national, mixed media dissemination campaign will be led by the National Community Pharmacists Association (NCPA) to convey recommendations to practicing pharmacists.

**Discussion:**

The guideline development process aims to incorporate the perspectives of multiple stakeholders and emphasize the importance of addressing the regulatory and pharmacy-specific aspects of care in addition to clinical evidence and guidance. The development of this guideline will provide targeted, multidisciplinary guidance for pharmacists, improving access to safe and effective opioid use disorder treatment in the community setting.

**Preregistration:**

This protocol was registered with the Open Science Framework in March of 2023. Registration may be found at: 10.17605/OSF.IO/6S9DY.

**Supplementary Information:**

The online version contains supplementary material available at 10.1186/s13690-024-01287-4.

**Table:** 

**Text box 1. Contributions to the literature**
• Less than half of all community pharmacies in the US stock buprenorphine products for the treatment of opioid use disorder.
• Conventional practice guidelines do not address administrative barriers that prevent pharmacies from stocking and dispensing buprenorphine.
• The Pharmacy Access to Resources and Medication for Opioid Use Disorder will leverage a modified Delphi design and a multidisciplinary expert panel to create pharmacist-oriented, consensus guidance to promote access to buprenorphine in community pharmacies.

## Introduction

There are currently only three medications indicated for the treatment of opioid use disorder (OUD) in the United States [1]. Of these, buprenorphine, a partial opioid agonist, is unique in that it may be dispensed from a community pharmacy pursuant to a multi-day prescription for self-administration. Historically, buprenorphine could only be prescribed by clinicians holding a Drug Abuse Treatment Act of 2000 (DATA-2000) waiver [2]. The passage of DATA-2000 and the institution of registration and training requirements was intended to improve quality of care for persons with opioid use disorder. Historically, training and registration requirements conflicted with the pressing need to expand the addition medicine workforce. Since the passage of DATA-2000, researchers and implementation scientists have focused most of their efforts on understanding and circumventing barriers to provider access [3–5]. 

The COVID-19 Pandemic introduced new challenges to traditional office-based encounters and heightened interest in further reducing barriers to provider access. This manifested in a newfound reliance on telehealth service delivery [6] and increased the intensity of advocacy efforts aimed at eliminating DATA-2000 registration requirements. Efforts were successful on both fronts. Telehealth buprenorphine treatment programs were shown to increase treatment retention by as much as 71% [6] and on December 30, 2022, the bipartisan Mainstreaming Addiction Treatment Act (MAT Act) eliminated DATA-2000 registration requirements providing a pathway for patients to access buprenorphine directly through a preferred or convenient prescriber [7]. While efforts to extend access to prescriber services represent true progress, little has been done to address another critical component of the medication use process: access to medication in community pharmacies [8]. 

Buprenorphine products indicated for the treatment of opioid use disorder are not universally available in community pharmacies. An audit of 5,734 pharmacies in 11 states found that only 48.3% actively stocked buprenorphine/naloxone combination products [9]. Across all states, 65.4% of independent pharmacies did not have buprenorphine/naloxone combination products in stock and less than half (47.5%) were willing to order it from their wholesaler. To understand the scope of these findings, it is necessary to understand the inherent complexity of community pharmacy practice. Controlled substance purchase and dispensation in community pharmacies is governed by a complex patchwork of state and federal regulations. In addition to state and federal regulations on pharmacy practice, the Drug Enforcement Administration (DEA) Suspicious Order Report System requires pharmaceutical wholesalers to report orders flagged as suspiciously large or otherwise suspicious [10]. The DEA has publicly stated that there are no fixed quantitative parameters that define a “suspicious” order. Rather, wholesalers are obligated to set their own parameters subjected to Drug Enforcement Administration review [10]. The lack of transparency in the suspicious order reporting process has led pharmacists to believe that fixed quantitative limits on wholesale buprenorphine purchase exist [11]. Available evidence suggests that pharmacists feel that purchasing buprenorphine, even in necessary quantities to provide care for persons with opioid use disorder, could compromise their ability to order other controlled substances or even lead to punitive action [12]. These claims are not entirely unwarranted. As of late 2022, American pharmacy corporations have settled for thirteen billion US Dollars in damages related to opioid dispensing [13]. While these settlements were not directly related to buprenorphine dispensing, buprenorphine is a Schedule III controlled substance and a partial opioid agonist. As such, pharmacy corporations have restricted buprenorphine dispensing alongside other opioids. Patients are currently paying the price for corporations’ failure to differentiate buprenorphine from other opioid analgesics.

The state of care for persons with opioid use disorder in community pharmacies contrasts with that put forth in prevailing practice guidelines from the Substance Abuse and Mental Health Services Administration (SAMHSA) which assume that all pharmacies dispense buprenorphine [1]. While the clinical recommendations and evidence available in the SAMHSA guidelines are sound, their omission of regulatory and pharmacy-specific aspects of care limit their applicability to pharmacy practice. A longstanding focus on diversion control has made it challenging for pharmacists to make dispensing decisions guided by healthcare quality and patient preference. Barriers to buprenorphine supply in community pharmacies is a tale of obstruction at all levels in the regulatory and drug supply chain. Addressing supply barriers, therefore, requires input from stakeholders at all levels including regulators, law enforcement, pharmacy corporations, prescribers, and practicing pharmacists. In this document, we describe a protocol for the creation of a multi-disciplinary, evidence-based practice guideline to support buprenorphine dispensing in community pharmacies. Unlike prevailing guidelines, this guideline will rely on input from pharmaceutical wholesalers, state boards of pharmacy, and law enforcement agencies, as well as clinicians, to generate targeted, multidisciplinary guidance for pharmacists improving access to safe and effective opioid use disorder treatment in the community setting.

## Methods

### Methodological overview

Pharmacy practice is governed by a wide array of clinical and regulatory stakeholders that often issue seemingly conflicting guidance and policy for practicing pharmacists. Clinical guidelines for the management of opioid use disorder provide a strong, clinical rationale for pharmacists to actively participate in treatment by dispensing buprenorphine and providing cognitive services, including counseling, to patients prescribed buprenorphine [1, 14, 15]. They do not, however, address areas of confusion at the interface of regulation, enforcement, and best clinical practice. The objective of the guideline development process detailed here goes beyond synthesizing clinical evidence to address the myriad factors that impact pharmacists’ ability to provide care for persons with opioid use disorder in their practice.

To address this need, this process will proceed in three phases following a technique closely aligned with the Institute of Medicine’s guidance on guideline development: *Clinical Practice Guidelines We Can Trust* [16]. In the first scoping phase, we will conduct a series of qualitative interviews with pharmacists in three states: Texas, California, and West Virginia to identify their salient attitudinal, behavioral, and normative beliefs toward buprenorphine dispensing [17]. The results of these interviews will set the scope of the guideline and the supporting literature review [16, 18]. We will next use a multidisciplinary, four-round modified Delphi panel to develop consensus recommendations to support buprenorphine dispensing in community pharmacies. After guideline creation, the National Association of Boards of Pharmacy will coordinate a public comment period and external expert review of the recommendations. Finally, the National Community Pharmacists Association (NCPA) will lead a national, mixed media dissemination campaign to convey recommendations to practicing pharmacists. A methodological overview of each of these phases is provided in the following sections.

### Role of the investigators

The authors of this protocol will be responsible for study oversight and will work together to complete analytic and reporting tasks throughout the two-year duration of the proposed research. Most of the study team members (TV, HF, DT, MW, LH, DD, JW, and DG) are licensed pharmacists with prior experience in the community (TV, HF, JW), ambulatory care (LH, DD), psychiatric (DD), and inpatient settings (DT, MW). Several have held executive leadership positions or employment in national pharmacy professional organizations including the National Association of Boards of Pharmacy (JB, JW), the National Community Pharmacists Association (HF) and the American Society of Health-Systems Pharmacists (DG). In addition to their professional credibility, the leadership team has sufficient methodological training and experience to complete the proposed research. JE is a highly experienced qualitative methodologist. TV and DT are PhD trained, mixed-methods health services researchers who focus on evaluating the quality of care for persons living with or at risk of developing opioid use disorder. DG holds a PhD in education administration and is a highly accomplished qualitative researcher. LH, DD, and MW each contribute their clinical practice and research experience in substance use disorder, healthcare quality, and behavioral health. All members of the study team have contributed to the development and revision of this protocol. The study team will continue to meet at least monthly through the duration of the guideline development phase as specified in this document.

### Qualitative elicitation and scoping

#### Theoretical framework

The theory of planned behavior holds that an individual’s intention of performing a behavior is grounded in their attitudinal, normative, and control beliefs toward the behavior. In Ajzen’s original framework, attitudinal beliefs refer to an individual’s beliefs about a behavior and their evaluation of those beliefs. Normative beliefs refer to the perceived views of relevant others toward a behavior [19]. Attitudinal beliefs and normative beliefs form the basis of Fishbein’s theory of reasoned action [20]. The theory of planned behavior expands on Fishbein’s original framework by incorporating perceived behavioral control or the level of difficulty associated with performing a behavior in the context of the external resource environment [19]. If it can be demonstrated that external factors may influence an individual’s intention to perform a behavior, then the theory of planned behavior provides a more accurate model of the behavioral process than the theory of reasoned action. This is the case in pharmacy practice, particularly in the controlled substance prescribing process, where pharmacists are subjected to significant influence from employers, federal and state law enforcement organizations, and patients to act [21, 22]. The TPB, therefore, provides an ideal framework to elucidate scenarios where addressing maladapted perceptions of the external resource environment or behavioral beliefs conflict with existing evidence based clinical guidance.

#### Qualitative elicitation

The ability to detect external control and normative influences has made the TPB an invaluable framework for qualitative measurement of pharmacists’ intentions to engage in patient care behavior. To set the scope of this practice guideline, we will conduct a series of focus groups with a purposeful sample of pharmacists in three states: Texas, California, and West Virginia. These states were selected largely for their varying opioid use disorder risk environment and resource allocation strategies. Each state is demographically and socioeconomically diverse. To ensure that an adequately representative sample of pharmacist beliefs is captured in the elicitation study, we will divide pharmacists, based on their publicly listed practice address, into four strata defined by the Area Deprivation Index [23, 24] and rurality [25]. A detailed rationale and review of the policy environment in each state and a detailed sampling strategy are provided in Appendix (A) Two participants from each stratum will be selected to participate in each eight-member, moderated focus group. The focus group format and moderator guide were developed using Sutton’s technique to elicit attitudinal, normative, and behavioral beliefs [22, 26–28]. The focus group moderator guide is provided in Appendix (B) In addition to classical TPB grounded, open ended items meant to elicit behavioral, normative, and control beliefs toward buprenorphine dispensing, the moderator guide contains a series of targeted, probing questions intended to elicit specific beliefs about wholesaler interactions, alarming prescription characteristics or “red flags”, patient characteristics, observed behavior of other pharmacy personnel, and perceived clinical appropriateness of long-term maintenance buprenorphine treatment. Each of these topics have been identified in earlier literature as potential barriers to buprenorphine dispensing in community pharmacies. Each focus group is expected to last approximately two hours. Pharmacist participants will receive an incentive valued at $200 for their participation. We expect to complete two focus groups in each state.

Focus groups will be transcribed by a professional, human transcription service. Transcribed focus group results will be analyzed using a reflexive thematic approach. The goal of the thematic analysis is to develop vignettes for the first round of the Delphi panel. After focus groups are completed, two researchers (JE, MW) will read and re-read the transcripts to establish familiarity with the results [29]. A single transcript will be chosen at random and the researchers will each code it individually before reconvening to establish an initial set of codes [29]. They will then each individually code the transcripts recording new codes as they emerge. After completing their individual analyses, the researchers will convene to review new codes and to group the identified codes into themes. The researchers will be encouraged to remain reflexive throughout the process but to pragmatically focus their analysis on the identification of practice scenarios that influence pharmacists’ attitudes and intentions to provide care for persons with opioid use disorder [29]. If the JE and MW cannot agree on the interpretation or definition of a theme, TV will serve as a referee. The themes will be grouped, as appropriate, to form a series of scenario-based vignettes that can be used to elicit appropriate actions in round one of the subsequent Delphi panel.

### Delphi panel

#### Constructing the evidence packet

After scoping is complete, we will conduct a systematic literature review to compile evidence for the Delphi panel. Clinical practice guidelines are only as trustworthy as their underlying literature review [16]. The qualitative results will guide the development of the search strategy and article inclusion criteria. Literature retrieval will be performed in three databases: PubMed, CINAHL, and the Cochrane Library. Two graduate research assistants and one researcher will review the extracted articles, remove duplicates, and screen for inclusion following criteria established at the conclusion of the qualitative elicitation study. Full text articles will be compiled into an evidence packet for distribution to Delphi panel members. The systematic review team (TV, JW, DG) will then compile an annotated bibliography that provides a neutral summary of the objectives and results of each included publication. This will be indexed according to the vignette or vignettes each publication is intended to support.

In this case, the scope of the guideline is expected to extend beyond clinical, peer reviewed literature and a traditional systematic review of the evidence restricted to clinical databases would be irrelevant. We will supplement the systematic review packet with a policy and regulatory review conducted by two contract consultants (JW and DG) with expertise in pharmacy regulatory affairs and controlled substance enforcement policy. Policy documents will be compiled, summarized, indexed according to vignette, and presented to experts along with the clinical evidence packet.

#### Assembly of the expert panel

Delphi panels are usually constructed through purposive sampling. The goal of our sampling strategy is to identify a panel of experts that represents a wide array of perspectives in pharmacy practice, managed care, state boards of pharmacy, and law enforcement organizations. After the scope of the guideline is defined and the initial vignettes are prepared, members of the study team will be contacted individually and asked to identify experts in each of the following four areas:

- Psychiatrists and psychiatric pharmacists with experience in the clinical management of opioid use disorder with outpatient buprenorphine.
- Current members of state boards of pharmacy.
- Current or former diversion control officers and administrators from within the Drug Enforcement Administration.
- Employees of pharmaceutical wholesalers or pharmacy services administrative organizations who have first-hand administrative involvement with suspicious order monitoring systems or diversion control policy and procedure.
- Independent community pharmacy owners and corporate pharmacy chain managers and executives.

The nominating member will be asked to provide a short summary of each potential participant’s qualifications for membership on the Delphi panel and a short description of their area of expertise. The remaining members of the study team will be asked to rate the appropriateness of the candidates in each area of expertise based on the nominator’s description. Study team members will be asked to abstain from rating their own nominees. The eight highest rated nominees in each of the above categories of expertise will be invited to join the expert panel and offered $1,000 honoraria for completing all four rounds of data collection. If potential participants decline, the next highest rated in that area of expertise will receive an invitation to participate. Our goal is to empanel 40 participants. We expect an attrition rate of 20% [30], leaving 32 participants to participate in round four, a sufficient number to produce stable consensus ratings free of the noise associated with larger panels [31]. Potential participants will be required to submit a standard conflict of interest disclosure prior to the initiation of data collection. The guideline generating committee will meet to review submitted conflicts. If a potential panelist reports a financial conflict of interest and an agreeable management plan cannot be determined or if divestment of interests is not feasible, that individual will be removed from the panel and an alternative panelist will be selected [16].

#### Round one

The objective of round one is to identify the range of responses to the vignettes defined by the qualitative elicitation study. The vignette responses will form the basis for the candidate consensus statements in rounds two through four. After agreeing to serve on the expert panel, participants will receive an electronic copy of the evidence and policy packets and the first-round data collection instrument. In the first round, participants will be provided with a series of vignettes that describe situational or attitudinal barriers identified by pharmacists during the elicitation study. After reading each vignette, the participant will be asked to do the following:

1. Rate the likelihood of adverse outcomes if a pharmacist was to act in each scenario, scored on a nine-point Likert type scaled anchored from “Extremely Unlikely” to “Extremely Likely”.
2. Identify potential actions that community pharmacists can take to avoid adverse outcomes of buprenorphine dispensing.
3. Identify potential actions that pharmacy owners and pharmacy corporations can take to avoid adverse outcomes of buprenorphine dispensing.
4. Suggest policy changes that would support the ability of pharmacists to provide buprenorphine.
5. Identify clinical evidence or regulatory guidance that influenced their response but was not included in the provided supporting material.

**Fig. 1:**
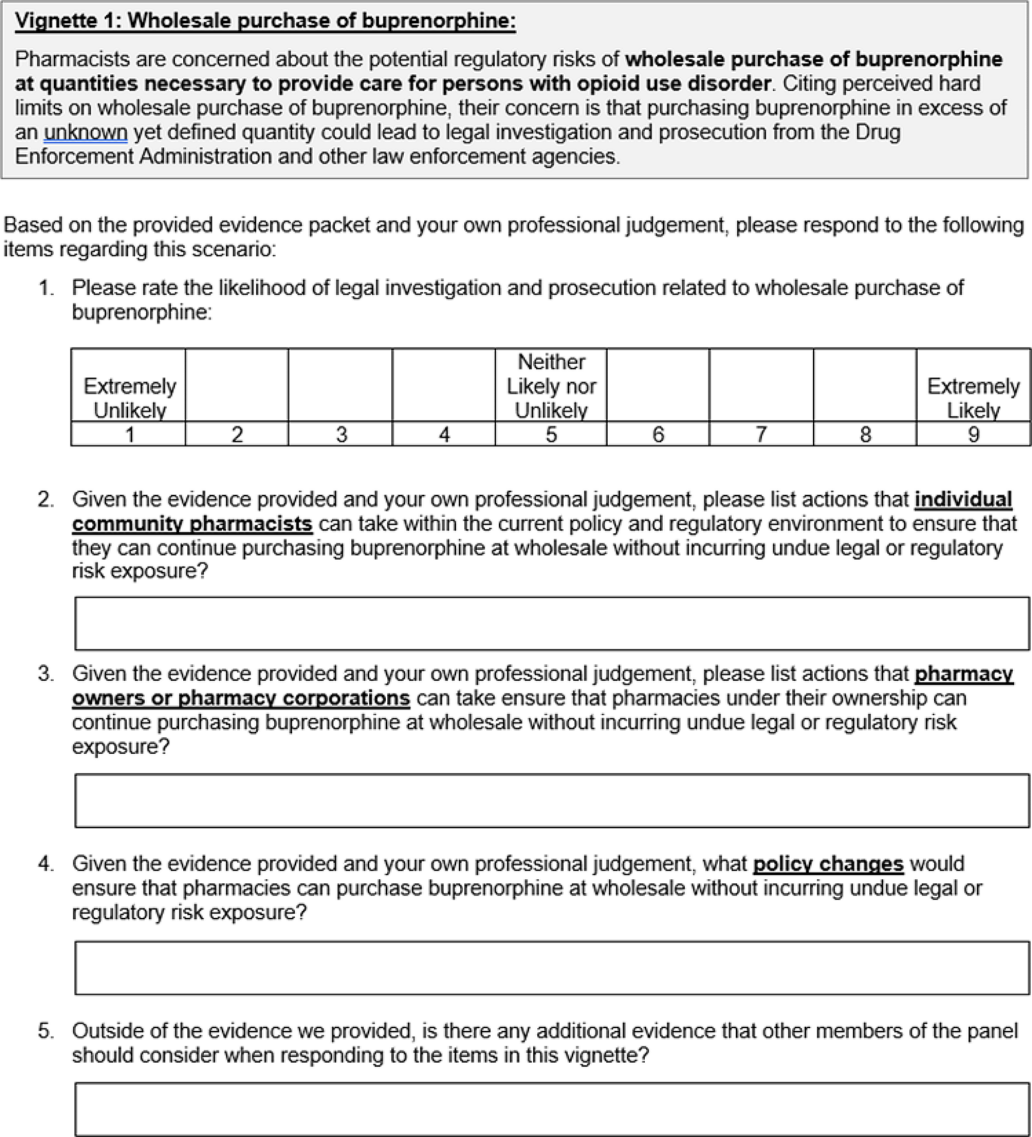
Example vignette that will be used to elicit candidate consensus statements in a Delphi study intended to create guidance for community pharmacists to increase availability of buprenorphine in the community setting

An example vignette is provided in Fig. 1. The vignettes will be presented to panelists as an online data collection instrument built in Qualtrics. Panelists will be provided three-weeks to complete their initial ratings. Weekly reminders will be sent to maximize retention.

After the vignette responses are elicited, two researchers will review the responses using an inductive, thematic approach. Each response to each of the four individual, open-ended items will be coded independently by each researcher assigned to this task. Similar proposed actions or solutions will be aggregated into themes and each independent code or theme will be retained into the phase-two data collection instrument. At this point, all members of the study team will be asked to review the draft statements generated in the first round and will meet to discuss the uniqueness and feasibility of each statement. Statements viewed as redundant or infeasible will be revised or removed. The removal of any statement and a description of rationale for removal during this initial content validation will be published alongside the final guideline.

#### Rounds two and three

In round two, participants will be provided with a new vignette-based instrument. Each vignette will now be presented alongside the proposed pharmacist, pharmacy chain, and policy actions proposed in round one. Participants will also be provided with a histogram summarizing the panel’s response to the quantitative likelihood of adverse consequences item from round one. The participant’s individual response will be indicated on the histogram. In their round two response, panelists will first be asked if they wish to revise their rating of the likelihood of adverse consequences from each vignette. A textbox will be provided to allow participants to justify their decision to revise or retain their initial rating considering other panelists’ responses. Next, participants will be asked to rate the appropriateness and effectiveness of each action on a nine-point Likert type scale anchored from “Not at all appropriate” to “Extremely appropriate” and “Not at all effective” to “Extremely effective”, respectively. After participants rate each item, they will also be provided a textbox and instructed that they may suggest revisions to the wording of the statement or provide rationale for their rating.

Panelists will be provided three-weeks to complete their round two ratings. After round 2, statements that 75% or more participants rated as seven or higher on the effectiveness and acceptability measures will meet the a-priori definition of consensus. Statements that 75% or more participants rated as three or lower on either the acceptability or effectiveness scales will be removed from future participation. At the conclusion of data collection, a member of the research team will catalogue suggested changes to the remaining items and prepare a report for the remainder of the research team. The research team will review, discuss, and implement suggested changes in the event that similar changes to the item were recommended by at least two panelists, erring on the side of original item wording so as to not inject bias [32]. Items that did not meet consensus or exclusion criteria will be retained in the round three instrument.

In round three, panelists will be provided with a packet summarizing the previous round. This packet will contain a list of the statements that reached consensus, a list of the statements that did not reach consensus, and a histogram summarizing the round two effectiveness and acceptability statements indicating their own response to each statement. The data collection instrument in round three will largely resemble that in round two. Panelists will be shown a summary of their peers’ round two ratings and comments on each remaining statement as well as their own response to the effectiveness and acceptability items. At this time, they will be provided the opportunity to revise or retain their prior rating and allowed to justify their decision in a free-text field.

Participants will be provided three-weeks to complete round three. The analysis at the end of round three will follow the procedure used at the conclusion of round two. The summary of rejected statements and statements that reached consensus will be updated and the remaining statements will be summarized in advance of the live round four discussion.

#### Round Four

In round four, we will convene a moderated, virtual, six-hour long workshop to debrief the panel on the guideline creation process and reassess items that were still indeterminant. The discussion will begin with a presentation from the study team intended to review the statements that reached consensus, review those that were rejected, and to review those that were still indeterminant. Three researchers, serving as a panel of moderators, will then lead a semi-structured discussion in which each remaining statement will be presented along with a description of the comments on that item in the previous rounds. The moderator will facilitate a timed discussion for each statement. The amount of time allocated for discussion will depend on the number of statements remaining. Immediately after each statement is discussed, participants will be asked to re-rate the effectiveness and acceptability of the action proposed in the statement. The ratings will be summarized in real time and disclosed to the panel.

#### Strategies to increase retention

At the time of recruitment, panelists will be informed that payment of the $1,000 honoraria is contingent on their completion of all four rounds of the Delphi panel. Participants who complete all four rounds will also be acknowledged by name in the final publication [18]. To ensure transparency, participants will be informed of the start-date and duration of each data collection period. They will receive a graphical timetable at the time of recruitment and electronic calendar invitations for key dates at the time of enrollment. Weekly reminders will be sent to stimulate responses during each response period. A structured panel design with a fixed number of rounds, transparency of process, and frequent reminders have all been shown to maximize retention in Delphi studies [32].

### Validation, External Review, and dissemination

The resulting practice guideline will be organized into three sections defined by recommendations for pharmacists, recommendations for pharmacy organizations, and recommendations for policy makers. The National Association of Boards of Pharmacy will facilitate a 60-day, online public comment period to allow relevant stakeholders, patients, and members of the public to suggest changes to the preliminary draft. A mixed-media communication campaign, led by the National Community Pharmacists Association and the National Association of Boards of Pharmacy, will announce the availability of the draft guidelines and the opening of the public comment period to key patient advocacy, corporate, regulatory, and professional stakeholders as well as the public at large. In addition to direct communication to key stakeholders, NABP will release a general press release announcing the creation of the guidelines and the opportunity for public comment approximately 30 days before the beginning of the public comment period. Both organizations will also leverage social media campaigns to encourage public participation. After the public comment period has concluded, the comments will be analyzed thematically [33]. The National Association of Boards of Pharmacy will then convene a panel of 10–15 experts who were not involved in the initial guideline creation process for an in-person meeting at their headquarters in Mount Prospect, Illinois. This panel will be recruited by the National Association of Boards of Pharmacy and will include experts in pharmacy regulatory affairs, law enforcement (e.g., the Drug Enforcement Administration), representatives of pharmacy wholesale organizations, representatives of community pharmacy corporations, and representatives of pharmacy trade associations. The panel will meet for a one-day workshop to review the aggregated comments and discuss potential changes to the guideline document. A report of the proceedings of this workshop will be reviewed by the study team. Changes suggested by the public and endorsed by the NABP panel will be accepted if the following criteria are met:

1. The individual or entity who initially proposed the change has no clear financial or material interest in the proposed modification.
2. A simple majority of research team members believe that incorporating the proposed change would benefit the safety and real-world effectiveness of treatment or otherwise clarify the process of care for practicing pharmacists who dispense buprenorphine.

The review process and the recruitment of a separate, but similar, expert panel by the sponsor organization is similar to the process used by the Centers for Disease Control and Prevention in the creation of the 2022 Clinical Practice Guidelines for Prescribing Opioids for Pain [34]. Recruiting a separate peer review panel is intended to foster an unbiased, rigorous review of public comments. After the conclusion of the public comment period and associated review, the research team will finalize the draft of the guidelines and initiate a national dissemination, education, and evaluation campaign. In this final phase of the research, the National Community Pharmacists Association will leverage targeted online advertising, email circulation, webinars, instructional videos, and accredited continuing pharmacy education to disseminate the guidelines to practicing community pharmacists. The reach and effectiveness of the campaign will be evaluated through a series of longitudinal surveys intended to measure changes in pharmacists’ attitudes toward and intention to dispense buprenorphine following educational engagement. The planned, multifaceted dissemination and evaluation campaign is complex and will be addressed in a future protocol.

#### Role of the funder

The Foundation for Opioid Response Efforts (FORE) is a private, not for profit, grant-making foundation focused on identifying and promoting solutions to the US opioid crisis. FORE will provide financial support through all phases of this body of work through an unrestricted grant to the University of Houston College of Pharmacy (Tyler Varisco, principal investigator). A program officer from FORE will represent the foundation as an ex-officio observer at steering committee meetings. FORE will assist with public relations, guideline dissemination, and education dissemination.

## Discussion

To conclude, this study will represent the first attempt to generate consensus practice guidelines on the care of patients with opioid use disorder in community pharmacies. The proposed research is important in that it goes beyond the clinical to capture critical regulatory and commercial factors that interfere with community pharmacists’ ability to provide care for persons with opioid use disorder. By relying on a multidisciplinary panel of experts from a variety of backgrounds, our primary goal is to identify areas where pharmacists’ perceptions of the practice environment contrast with the well-defined clinical need to provide treatment.

In 2019, only 27.8% of the 2.2 million individuals with active opioid use disorder in the United States reported past-year use of medication for opioid use disorder [35]. One of the most salient barriers to treatment remains difficulty accessing and maintaining access to medication for opioid use disorder in community pharmacies [9, 36, 37]. By providing tailored guidance to pharmacists and incorporating perspectives rarely captured in clinical practice guidelines, our goal is to not only clarify pharmacists’ scope of practice and obligation to patients with opioid use disorder but to also provide recommendations to policy makers and researchers to continue to maximize the clinical value of pharmacist services for patients with opioid use disorder. The Mainstreaming Addiction Treatment Act extends access to prescriber services, but little progress has been made to ensure that issued buprenorphine prescriptions can be dispensed from any community pharmacy. Achieving that goal will require targeted guidance, education, and intervention and this rigorous, trustworthy, and transparent practice guideline is expected to provide a clear path to achieve that goal.

### Electronic supplementary material

Below is the link to the electronic supplementary material.

Supplementary Material 1

Supplementary Material 2

## Data Availability

No datasets were generated or analysed during the current study.
