# Supplementary material for: Protocol for the development of a consensus practice guideline To address clinical and regulatory barriers to buprenorphine dispensing in community pharmacy
Source: Arch Public Health. 2024 Apr 25;82:58. doi: 10.1186/s13690-024-01287-4 (PMC11044383; doi:10.1186/s13690-024-01287-4)
Supplement: Supplementary file 1 — Supplementary Material 1 [file 13690_2024_1287_MOESM1_ESM.docx]

***Appendix A: Sampling Strategy***

**Rationale for sampling strategy:**

In the planned elicitation study, we will conduct six, virtual focus groups of eight community pharmacists each in three states: Texas, California, and West Virginia to conceptualize factors associated with pharmacists’ intention to dispense buprenorphine. These three states were selected in that they each vary in terms of need, resource availability, approach to delivery, and policy context. California has effectively leveraged Medicaid funding to expand access to treatment for opioid use disorder. Through Medicaid expansion, the clever implementation of Medicaid Section 1115 Demonstration projects and (SOR) funding, California has been able to rapidly expand access to care for high-risk individuals through the implementation of extended service delivery in low-access areas(1). David Dadiomov, the California site-PI, is the clinical coordinator for the Whole Person Care pharmacy program in Los Angeles County. This Section 1115 pilot program (2) leverages a variety of innovative, pharmacy service models, including mobile pharmacy services, to extend access to buprenorphine. We expect that the perspective of pharmacists from a well-resourced and progressive state will help to identify access facilitators which are of equal importance to barriers.

Demographically, Texas is similar to California in that it is a populous and diverse state with concentrated urban areas and sparse rural areas. The resource environment in Texas, however, contrasts sharply with that in California. First and foremost, Texas did not expand Medicaid under the provisions of the affordable care act. Section 1115 spending in Texas has mainly centered on inpatient and managed care projects with little attention paid to substance use disorder. While Texas does receive SOR funding, SOR funded programs in the treatment domain have focused predominantly on the development of bridge programs and DATA 2000 waiver training. In the absence of resource investment, only 42.2% of all community pharmacies dispense buprenorphine.(3). We expect that elicitation focus groups in Texas will provide a revealing exposition of structural, as well as behavioral and attitudinal barriers, to buprenorphine dispensing.

West Virginia is a Medicaid expansion state and has used SOR funding to dramatically expand access to treatment through the development of standardized hub and spoke programs statewide.(4) Our earlier work from 2017 suggests that buprenorphine is also more widely available in West Virginia community pharmacies with 74.8% reporting supply on hand.(5). With 3.2% of the population living with opioid use disorder, demand for buprenorphine in West Virginia is notably higher than that in other states. Still, treatment capacity still lags despite intensive resource investment.(6) Earlier research has found that provider stigma and regulatory barriers may be responsible for the gap in capacity but the pharmacists’ perspective remains almost absent from the literature. Dr. Scott and Dr. Thornton have worked extensively with the West Virginia State Board of Pharmacy and are both familiar with pharmacy practice in that state. They will leverage his deep ties to the profession of pharmacy in West Virginia to recruit pharmacists for the proposed focus groups. Elicitation work in these three, politically and demographically diverse states will provide significant insight into how pharmacists’ attitudinal, normative, and control beliefs vary in the context of the resource and risk environment. Multistate, theoretically grounded qualitative work will provide a solid foundation for guideline development and future quantitative behavioral research.

**Sampling strategy:**

To capture the diversity of barriers and facilitators to buprenorphine dispensing, our goal is to recruit pharmacists in a way that addresses the scope of the pharmacy practice and risk environments within and between states. To this end, we will use a stratified purposeful sampling technique to recruit pharmacists from areas that vary in socioeconomic status and rurality within each state. This is consistent with the construction of focus groups aimed at establishing breadth rather than depth of an issue and is intended to facilitate the development of a scoping list of statements for the Delphi panel.(7, 8)

All three states, Texas, California, and West Virginia maintain registries of licensed pharmacists practicing in their state. Each registry contains contact information and information on each pharmacists’ place of employment. Licensed community pharmacists in each state will be categorized, based on their employment address, into one of four strata defined by area-level socioeconomic status and rurality. The low socioeconomic/rural strata will consist of pharmacists working in areas with an Area Deprivation Index(9-11) score in the sixth through tenth state-wide decile rank (most deprivation) and a United States Department of Agriculture Rural/Urban Communing Area (RUCA)(12) classification of “rural” or “small town” (RUCA 7-10) Conversely, the high socioeconomic/urban strata will be defined by an area deprivation index score in the first through fifth state-wide decile ranks (least through median deprivation) and a RUCA classification of metropolitan through micropolitan. Two quartiles representing urban/low socioeconomic status and rural/high socioeconomic status will also be defined.

After eligible pharmacists in each state are stratified, we will randomly select 25 pharmacists employed at independent community pharmacies and 25 employed at chain community pharmacies from each strata and assign a random number to each selected. A graduate research assistant or a member of the investigative team will then contact each pharmacist by telephone at their place of employment, inform them that they were selected to participate in a research focus group, and ask them if they have ever received a buprenorphine prescription. If they indicate that they have received a prescription for buprenorphine, regardless of whether they dispensed the prescription, they will be offered the opportunity to participate in a scheduled, incentivized focus group study. Two attempts will be made to contact and screen each pharmacist before moving to the next in the list of 25. Each focus group will contain eight pharmacists, two from each demographic strata. One focus group will consist entirely of community pharmacists employed at chain pharmacies and the other will consist entirely of independent community pharmacists.

This process is intended to adequately capture the extent of the practice environments in each state. In Texas, this process will lead us to sample pharmacists from the under-resourced and highly rural, Hispanic speaking Rio Grande Valley region as well as those practicing in the heart of our racially and economically diverse major metropolitan areas. In West Virginia, this will lead us to recruit pharmacists from well-resourced areas, including Morgantown, as well as rural areas in Southern West Virginia, arguably the heart of the opioid epidemic. Finally, in California, we will capture participants in Los Angeles and San Francisco as well as those in rural, wealthier, conservative areas. We expect the OUD access environment to vary significantly within strata and for this purposeful mixing of participants in diverse practice areas within focus groups to lead to the emergence of a broad array of potential attitudinal beliefs as well as barriers and facilitators to buprenorphine dispensing to be explored in the next phase of the research. The investigators at each partner college of pharmacy have extensive experience sampling participants within their respective states and Dr. Thornton has done extensive pharmacy practice research in West Virginia. As investigators from nationally respected colleges of pharmacy, we do not expect to have difficulty engaging with the pharmacy workforce to complete the proposed research.(5, 13-16)

**Supplemental Bibliography:**

1. Hinde JM, Mark TL, Fuller L, Dey J, Hayes J. Increasing Access to Opioid Use Disorder Treatment: Assessing State Policies and the Evidence Behind Them. J Stud Alcohol Drugs. 2019;80(6):693-7. Epub 2019/12/04. doi: 10.15288/jsad.2019.80.693. PubMed PMID: 31790360; PubMed Central PMCID: PMCPMC6900991.

2. Chuang E, Pourat N, Haley LA, O’Masta B, Albertson E, Lu C. Integrating Health And Human Services In California’s Whole Person Care Medicaid 1115 Waiver Demonstration: An overview of a California demonstration program focused on improving the integrated delivery of health, behavioral health, and social services for certain Medicaid beneficiaries. Health Affairs. 2020;39(4):639-48.

3. Hill LG, Loera LJ, Torrez SB, Puzantian T, Evoy KE, Ventricelli DJ, et al. Availability of buprenorphine/naloxone films and naloxone nasal spray in community pharmacies in 11 U.S. states. Drug and alcohol dependence. 2022;237:109518. doi: <https://doi.org/10.1016/j.drugalcdep.2022.109518>.

4. Winstanley EL, Lander LR, Berry JH, Mahoney JJ, Zheng W, Herschler J, et al. West Virginia's model of buprenorphine expansion: Preliminary results. Journal of Substance Abuse Treatment. 2020;108:40-7. doi: 10.1016/j.jsat.2019.05.005.

5. Thornton JD, Lyvers E, Scott VGG, Dwibedi N. Pharmacists' readiness to provide naloxone in community pharmacies in West Virginia. J Am Pharm Assoc (2003). 2017;57(2s):S12-S8.e4. Epub 2017/02/07. doi: 10.1016/j.japh.2016.12.070. PubMed PMID: 28163027; PubMed Central PMCID: PMCPMC5352525.

6. The Urban Institute. Do States and Counties Have Capacity to Treat Opioid Use Disorder? Analyses for DC, Maine, Michigan, New Jersey, New Mexico, Pennsylvania, and West Virginia. 2021.

7. Morgan DL. Focus groups as qualitative research: Sage publications; 1996.

8. Palinkas LA, Horwitz SM, Green CA, Wisdom JP, Duan N, Hoagwood K. Purposeful Sampling for Qualitative Data Collection and Analysis in Mixed Method Implementation Research. Adm Policy Ment Health. 2015;42(5):533-44. Epub 2013/11/07. doi: 10.1007/s10488-013-0528-y. PubMed PMID: 24193818; PubMed Central PMCID: PMCPMC4012002.

9. Kind AJ, Jencks S, Brock J, Yu M, Bartels C, Ehlenbach W, et al. Neighborhood socioeconomic disadvantage and 30-day rehospitalization: a retrospective cohort study. Ann Intern Med. 2014;161(11):765-74. Epub 2014/12/02. doi: 10.7326/m13-2946. PubMed PMID: 25437404; PubMed Central PMCID: PMCPMC4251560.

10. Knighton AJ, Savitz L, Belnap T, Stephenson B, VanDerslice J. Introduction of an Area Deprivation Index Measuring Patient Socioeconomic Status in an Integrated Health System: Implications for Population Health. EGEMS (Wash DC). 2016;4(3):1238. Epub 20160811. doi: 10.13063/2327-9214.1238. PubMed PMID: 27683670; PubMed Central PMCID: PMCPMC5019337.

11. Singh GK. Area deprivation and widening inequalities in US mortality, 1969-1998. American journal of public health. 2003;93(7):1137-43. Epub 2003/07/02. doi: 10.2105/ajph.93.7.1137. PubMed PMID: 12835199; PubMed Central PMCID: PMCPMC1447923.

12. Rural Health Research Center. The University of Washington School of Medicine Regional Medical Education Program. Zip Code RUCA Approximation. <https://depts.washington.edu/uwruca/ruca-approx.php>.

13. Fleming M, Bapat S, Varisco T. Using the theory of planned behavior to investigate community pharmacists' beliefs regarding engaging patients about prescription drug misuse. Res Social Adm Pharm. 2019;15(8):992-9. Epub 2018/11/18. doi: 10.1016/j.sapharm.2018.10.027. PubMed PMID: 30442574.

14. Evoy KE, Groff L, Hill LG, Godinez W, Gandhi R, Reveles KR. Impact of student pharmacist-led naloxone academic detailing at community pharmacies in Texas. J Am Pharm Assoc (2003). 2019. Epub 2019/11/02. doi: 10.1016/j.japh.2019.09.007. PubMed PMID: 31669417.

15. Hill LG, Loera LJ, Evoy KE, Renfro ML, Torrez SB, Zagorski CM, et al. Availability of buprenorphine/naloxone films and naloxone nasal spray in community pharmacies in Texas, United States. Addiction.n/a(n/a). doi: 10.1111/add.15314.

16. Dadiomov D, Bolshakova M, Mikhaeilyan M, Trotzky-Sirr R. Buprenorphine and naloxone access in pharmacies within high overdose areas of Los Angeles during the COVID-19 pandemic. Harm Reduction Journal. 2022;19(1). doi: 10.1186/s12954-022-00651-3.
