# Supplementary material for: Protocol for the development of a consensus practice guideline To address clinical and regulatory barriers to buprenorphine dispensing in community pharmacy
Source: Arch Public Health. 2024 Apr 25;82:58. doi: 10.1186/s13690-024-01287-4 (PMC11044383; doi:10.1186/s13690-024-01287-4)
Supplement: Supplementary file 2 — Supplementary Material 2 [file 13690_2024_1287_MOESM2_ESM.docx]

***Appendix B: Focus Group Moderator Guide***

**General Guidance for Moderator:** The entire discussion should be limited to two hours. If participants become distracted or the conversation goes off topic, return to the guide using gentle and courteous phrases like “I don’t want to take up too much of your time” or “I want to be cognizant of your time.” If participants seem to be providing new information, however, feel free to allow them to talk. Remember, the questions on this guide are meant to probe-not to lead. The pharmacists are the experts, and it is important to allow them to express their thoughts without coaching or leading. The focus group will be recorded and professionally transcribed so there is no need to have them repeat anything so you can take notes. You’re here to listen and interact. Not record.

**Introduction:**

“*Hello, thank you for joining us today to discuss your experience dispensing buprenorphine to persons with opioid use disorder. This is part of a series of focus groups that will be used to lay the foundation for professional practice guidelines to provide answers to common questions pharmacists have about the care of patients with opioid use disorder. There are no right or wrong answers to any of the questions we ask today, we just want to know what you’re doing now and where you think things could be improved through better guidance. Our conversation today will be recorded and quotes may be used in an upcoming academic publication. If you are quoted directly, your name, personal information, and contact information will remain completely anonymous. If anyone is not comfortable participating, you may drop off the call now. Otherwise, I’d like to give everyone an opportunity to introduce themselves and we can get started.”*

***Primary Questions (Theory of Planned Behavior)***

1. What do you believe are the advantages of dispensing buprenorphine for the treatment of opioid use disorder? (*Behavioral beliefs*)
2. What do you believe are the disadvantages of dispensing buprenorphine for the treatment of opioid use disorder? *(Behavioral beliefs*)
3. What individuals or groups would approve of you dispensing buprenorphine for the treatment of opioid use disorder? *(Normative beliefs)*
4. What individuals or groups would disapprove of you dispensing buprenorphine for the treatment of opioid use disorder? (*Normative beliefs)*
5. What circumstances enable you to dispense buprenorphine for the treatment of opioid use disorder? (*Control beliefs)*
6. What circumstances would make it more difficult for you to dispense buprenorphine for the treatment of opioid use disorder (*Control Beliefs)*

***Supplemental Questions (Others may be added as focus groups progress)***

1. How do you feel about ordering buprenorphine from your wholesaler?
2. Are there any specific scenarios where you are not comfortable dispensing buprenorphine?
3. If you had a patient that you thought would benefit from treatment, would you feel comfortable referring them to an outpatient treatment program? If so, walk me through your process.
4. To you, how long should patients receive medication for opioid use disorder?
5. Are their certain groups of patients that may have an easier time filling their buprenorphine prescription than others? More difficult?
6. Have you ever witnessed another pharmacist, technician, or staff member mistreat or inappropriately deny care to a person seeking treatment with buprenorphine? Can you tell us about that experience and your response?
7. Do you think your pharmacy is a welcoming environment for people seeking treatment for opioid use disorder? If so, what should other pharmacies do to be more open and welcoming for persons in treatment?
